# Long noncoding RNA *SFTA1P* promoted apoptosis and increased cisplatin chemosensitivity via regulating the hnRNP-U-GADD45A axis in lung squamous cell carcinoma

**DOI:** 10.18632/oncotarget.22138

**Published:** 2017-10-27

**Authors:** Ling Li, Ji-Ye Yin, Fa-Zhong He, Ma-Sha Huang, Tao Zhu, Yuan-Feng Gao, Yi-Xin Chen, Dong-Bo Zhou, Xiang Chen, Lun-Quan Sun, Wei Zhang, Hong-Hao Zhou, Zhao-Qian Liu

**Affiliations:** ^1^ Department of Clinical Pharmacology, Xiangya Hospital, Central South University, Changsha 410008, P. R. China; ^2^ Institute of Clinical Pharmacology, Hunan Key Laboratory of Pharmacogenetics, Central South University, Changsha 410078, P. R. China; ^3^ Department of Gerontology, Xiangya Hospital, Central South University, Changsha 410008, P. R. China; ^4^ Department of Dermatology, Xiangya Hospital, Central South University, Changsha 410008, P. R. China; ^5^ Center for Molecular Medicine, Xiangya Hospital, Key Laboratory of Molecular Radiation Oncology of Hunan Province, Central South University, Changsha 410008, P. R. China

**Keywords:** lung squamous cell carcinoma, long noncoding RNA SFTA1P, hnRNP-U- GADD45A, apoptosis, cisplatin chemosensitivity

## Abstract

Chemotherapeutic insensitivity remains one of the major obstacles in clinical treatment of lung squamous cell carcinoma (LSCC). Recently, increasing evidence has suggested that long non-coding RNAs (lncRNAs) promote tumorigenesis in many cancer types. However, the potential biological roles and regulatory mechanisms of lncRNAs in response to cisplatin treatment are poorly understood. Here, we found that lncRNA *SFTA1P* (surfactant associated 1, pseudogene), highly expressed in lung, was down-regulated in LSCC tissues and could be induced upon cisplatin treatment in LSCC cells. Elevated *SFTA1P* induced apoptosis and enhanced the sensitivity to cisplatin of LSCC cells. We further identified that hnRNP-U (heterogeneous nuclear ribonucleoprotein U) was down-regulated in LSCCs and positively correlated with patients’ poor prognosis as well as SFTA1P. Mechanistic studies revealed that *SFTA1P* could up-regulate hnRNP-U expression. In addition, we identified that hnRNP-U enhanced cisplatin-induced apoptosis through up-regulation of GADD45A, high expression of which was correlated with good prognosis in LSCC patients. Our findings demonstrated that *SFTA1P* might serve as a useful biomarker for LSCC diagnosis and a predictor for cisplatin chemotherapy response in patients with LSCC.

## INTRODUCTION

According to the GLOBOCAN project of the World Health Organization in 2012, lung cancer remains the top cancer killer worldwide (http://globocan.iarc.fr/). Lung squamous cell carcinoma (LSCC) is the second most common histologic subtype of lung cancer (preceded only by adenocarcinoma), accounting for 40∼50% of all primary lung cancers [1]. As opposed to adenocarcinoma, genomic alterations in LSCC have not been comprehensively characterized and no specific molecularly targeted therapies have been developed for the treatment of LSCC [1]. And unfortunately, targeted agents developed for lung adenocarcinoma are largely ineffective against LSCC. Currently, surgical resection is the primary treatment approach for early-stage LSCC [2]. While in locally advanced unresectable or metastatic LSCC, cisplatin-based chemotherapy remains the cornerstone of the front-line systemic treatment [3, 4]. Despite multiple options for the treatment of LSCC, the overall 5-year survival rate has not been improved significantly [5]. Chemotherapy resistance, either intrinsic or acquired, is a major clinical problem in LSCC, owing to individual variations in response to cisplatin based chemotherapy [3]. Therefore, understanding the molecular mechanisms underlying tumorigenesis and chemotherapy resistance is crucial for the development of effective therapeutic strategies for LSCC.

Long non-coding RNAs (lncRNAs) are transcripts longer than 200 nucleotides [8] which were once considered as transcriptional ‘‘noise’’ without biological function [6, 7]. However, the definition of lncRNA is controversial, recent research has shown that some lncRNAs can encode short peptides [9]. Accumulating evidence indicates that the majority of lncRNAs play key roles in cancer therapy through influencing cell cycle regulation, survival, cheomothrapy response, and various biological processes by modulating gene expression at the transcriptional, posttranscriptional and epigenetic regulation levels [10–14]. The majority (∼78%) of lncRNAs are characterized by their tissue-specific, developmental stage specific expression mode, in contrast to expression of mRNAs with only ∼19% of them with tissue specificity [15, 16]. Dysregulation of lncRNAs contributes to cancer progression, and they are therefore considered as potential therapeutic targets [17]. Our previous study found that deregulation of a panel of lncRNAs was involved in LSCC, based on a high throughput microarray screening [18]. Among them, *SFTA1P* was identified significantly downregulated in LSCC tissues, as compared with paired adjacent normal tissues.

In the current study, we focused on the function and regulatory mechanism of *SFTA1P* in LSCC. *SFTA1P* is a pseudogene-derived long non-coding RNA. By analyzing the relationship between *SFTA1P* and LSCC patients’ clinical features and survival, we found that decreased *SFTA1P* expression was correlated with poor prognosis. Further mechanistic studies revealed that *SFTA1P* could bind with and upregulate hnRNP-U. HnRNP-U, also named scaffold attachment factor (SAF)-A is involved in mRNA processing and transporting [19–21]. For instance, hnRNP-U can enhance the expression of GADD45A by stabilizing mRNA [20]. More recent studies have documented that hnRNP-U was involved in cell apoptosis [22], DNA damage response [23]. In this study, we identified that *SFTA1P* enhanced cisplatin-induced apoptosis through regulation of the hnRNP-U-GADD45A pathway. *SFTA1P* is an essential regulator in cisplatin induced apoptosis, and the *SFTA1P*-hnRNP-U-GADD45A signaling axis plays an important role in increasing LSCC chemosensitivity. In addition, knockdown of hnRNP-U also contributes to cisplatin resistance through decreasing the expression of the apoptosis and DNA repair related gene GADD45A, similar to *SFTA1P* depletion induced effects. Thus, we concluded that the lncRNA *SFTA1P* may enhance cisplatin sensitivity in LSCC by increasing the expression of hnRNP-U and further facilitating GADD45A expression.

## RESULTS

### Characterization of long non-coding RNA *SFTA1P*

**Figure 1:**
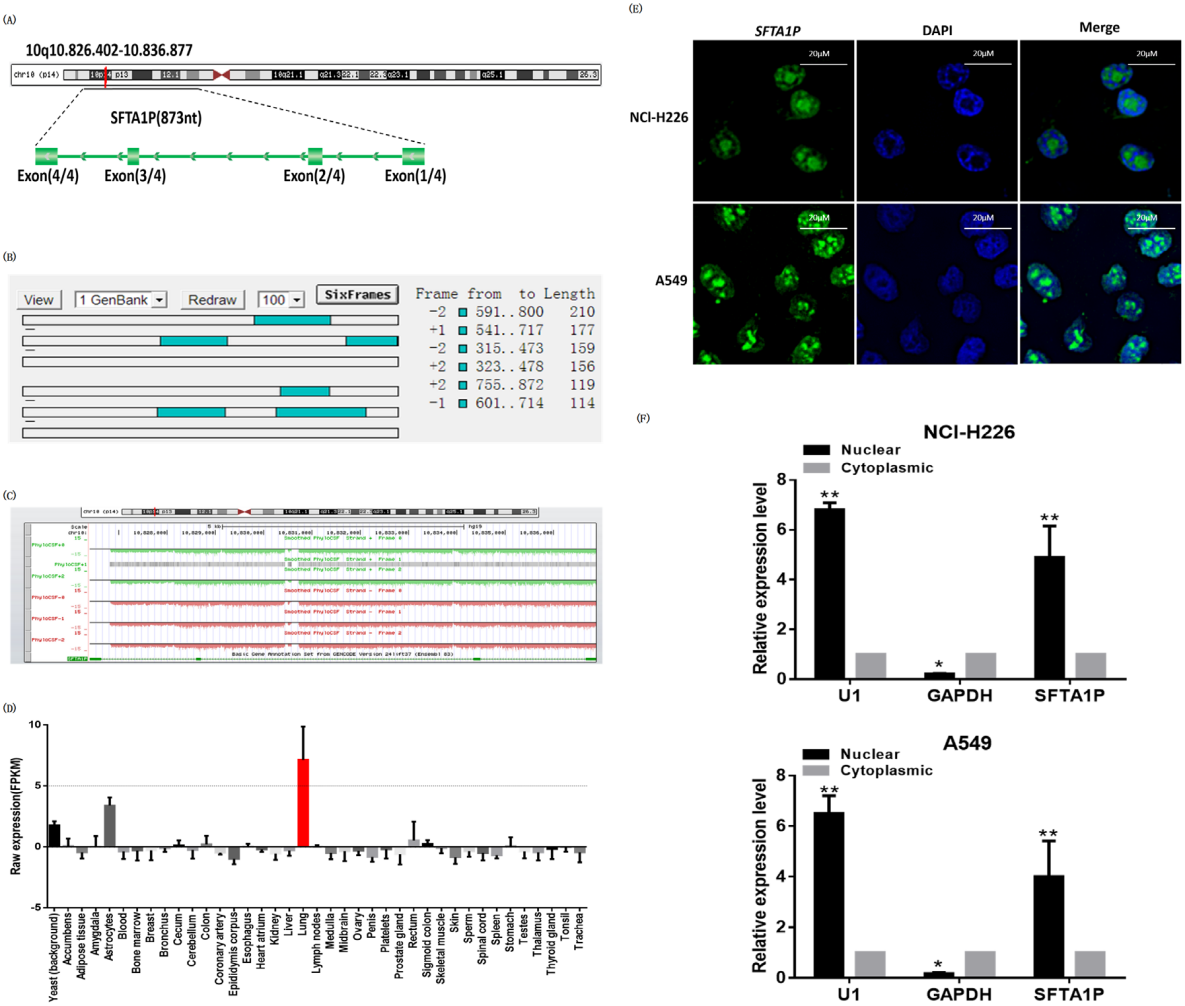
Characterization of *SFTA1P* **Panel A.** Schematic annotation of *SFTA1P* genomic locus on chromosome 10q10.826.402-10.836.877 in humans. Green rectangles represent exons. **Panel B** and **Panel C.** *SFTA1P* coding function analysis. Panel B. Analysis of the sequences by ORF Finder from the National Center for Biotechnology Information. Panel C. Used txCdsPredict from UCSC and PhyloCSF to calculate its coding potential. **Panel D.** SFTA1P specific expression in lung tissue. **Panel E.** Confocal FISH images showing nucleus localization of *SFTA1P* in NCI-H226 and A549 cells. Green represents *SFTA1P*; blue represent nucleus. **Panel F.** The RNA level of *SFTA1P* in nuclear and cytoplasmic fraction was determined by RT–PCR inNCI-H226 and A549 cells, respectively. U1 was a positive control for nuclear fraction and GAPDH was a positive control for cytoplasmic fraction. All experiments were performed in three biological replicates (n=3). And all data are shown as mean ±SD, ^*^p<0.05, ^**^p<0.05).

Long non-coding RNA *SFTA1P* is poly (A)-negative and located on chromosome 10 in humans. It consisted of four exons, with a full length of 873nt (Figure 1A). Its coding-potential was evaluated by analysis of the sequences using ORF Finder from the National Center for Biotechnology Information and it failed to predict a protein of more than 58 amino acids (Figure 1B). Moreover, lncRNA *SFTA1P* does not contain a valid Kozak sequence. In addition, we used txCdsPredict from UCSC and PhyloCSF [24] to calculate its coding potential, it further supported that lncRNA *SFTA1P* has very little protein-coding potential (Figure 1C).

It is well known that the subcellular localization will affect the lncRNA function in cells. Next, we employed an established fluorescence *in situ* hybridization (FISH) assay to observe *SFTA1P* localization in NCl-H226 and A549 cells by confocal laser scanning microscope. We found that *SFTA1P* was mainly located in the nucleus in both cell lines (Figure 1E). And this result was further verified by cytoplasmic and nuclear RNA fractionation analysis (Figure 1F). More importantly, according to RNA-Seq gene expression profiles from BioGPS Database [25], we found that lncRNA *SFTA1P* was specifically highly expressed in the lung (Figure 1D).

### The low expression of LncRNA *SFTA1P* was correlated with poor prognosis in LSCC patients

**Figure 2:**
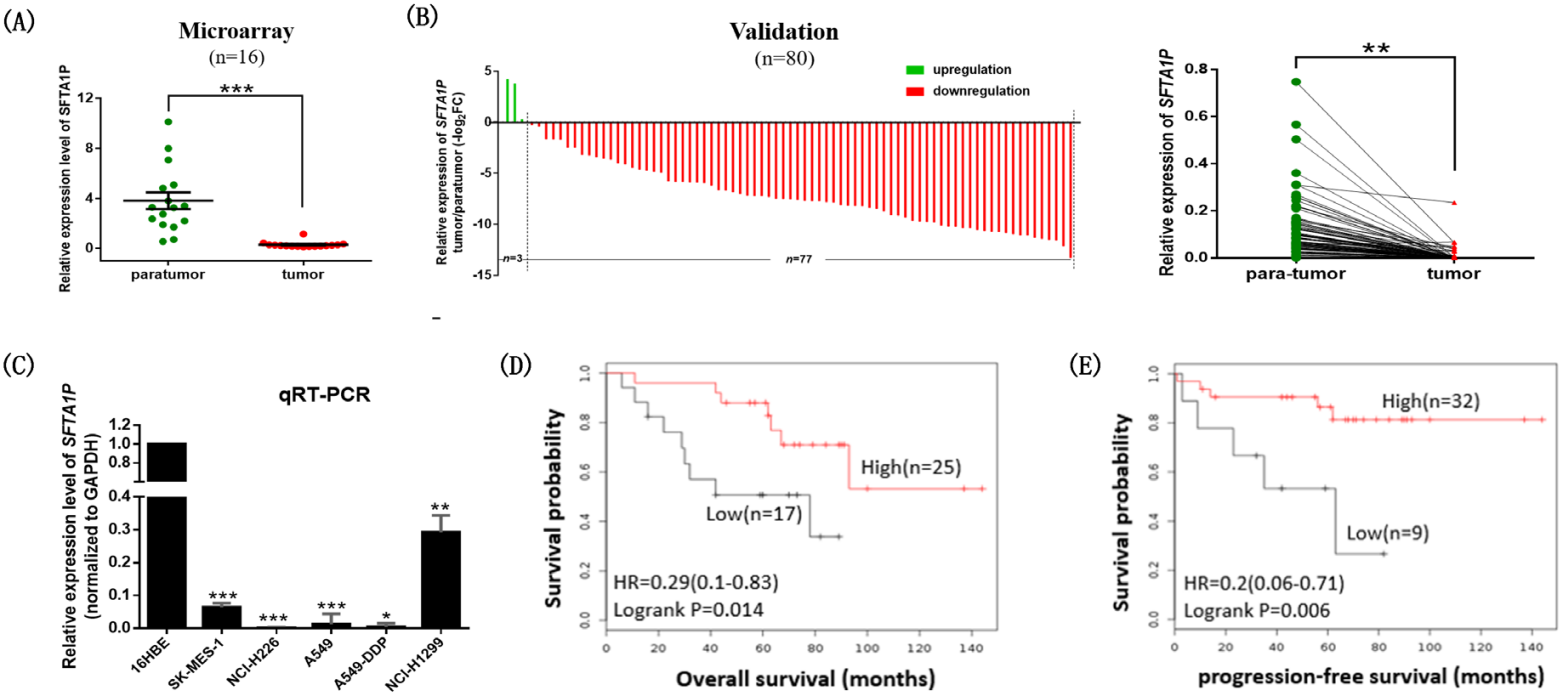
The expressions of LncRNA *SFTA1P* in LSCC cell lines and tissues as well as its potential clinical significance **Panel A-D.** Relative expressions of the *SFTA1P* in LSCC tissues and cells and its clinical significance. Panel A. The expression level of *SFTA1P* in LSCC patient‘s para-tumor and tumor tissues was determined by microarray (n=16). Panel B. The expression level of *SFTA1P* was independent validation in 80 LSCC patient’s para-tumor and tumor tissues by qRT-PCR. Panel C. qRT-PCR analysis of *SFTA1P* expression level in a normal human bronchial epithelial cell HBE and five common human NSCLC cell lines, NCl-H226, SK-MES-1, H1299, A549 and A549-DDP (cisplatin resistance cell line). ^*^p<0.05, ^**^p<0.01, ^***^p<0.001. **Panel** D-**E.** Kaplan-Meier survival curve of LSCC patients with low levels of *SFTA1P* expression were correlated with a poor (D)overall survival and (E)progression-free survival. The median *SFTA1P* expression was used as a cutoff (data from GSE50081).

In our previous study [18], we found that *SFTA1P* was downregulated in 16 LSCC tissues than in the paired adjacent normal tissues detected by microarray (Figure 2A). The result was further validated by qRT-PCR in another independent cohort of 80 paired LSCC tissues (Figure 2B). In addition, we analyzed the *SFTA1P* expression level in common LSCC cell lines (NCl-H226, SK-MES-1), lung adenocarcinoma (LUAD) cell lines (NCl-H1299, A549, A549-DDP) and a normal bronchial epithelial cell line (16HBE). Also, we found that *SFTA1P* was significantly down-regulated in both LSCC and LUAD cell lines than in 16HBE cells (Figure 2C). Of note, we found decreased expression of *SFTA1P* in A549 cisplatin resistant cells (A549-DDP) than in the parental A549 cell line (Figure 2C). These data indicated that *SFTA1P* may play a role in tumorigenesis and cisplatin sensitivity in lung cancer.

Meantime, we further examined whether *SFTA1P* expression was correlated with LSCC patients’ clinical pathological features using univariate analysis. The patients were divided into two groups: the high-*SFTA1P* group (n=40) and the low-*SFTA1P* group (n=40), using the median of *SFTA1P* as the cutoff. Statistical analysis revealed that decreased *SFTA1P* expression was correlated with advanced pathological stage (OR=2.38 (0.91, 6.26), p=0.077) and smoking status (OR=5.22 (1.46, 18.68), p=0.011). However, *SFTA1P* expression was not associated with other factors including age (OR=0.98 (0.92, 1.05), p=0.648), gender (OR=1.41 (0.07, 28.51), p=0.819), differentiation (OR=0.726 (0.13, 3.97), p=0.712; OR=0.802 (0.22, 2.86), p=0.734) and lymph node metastasis (OR=1.34 (0.51, 3.05), p=0.555) in LSCC (Table 1). Next, Kaplan–Meier survival analysis was performed to explore the relationship between *SFTA1P* expression and LSCC patient’s survival based on data from a GEO dataset (GSE50081). The results showed that the overall survival (OS) and progression-free survival (PFS) rate over 10 years for the low-*SFTA1P* group is shorter than that in the high-*SFTA1P* group (Figure 2D and 2E). These results demonstrated that lncRNA *SFTA1P* may serve as a biomarker to predict the prognosis of LSCC patients.

**Table 1:** Correlation between *SFTA1P* expression and clinicopathological characteristics in 80 LSCC patients

Clinical and pathological variables	N(%)	*SFTA1P* expression levels		*OR(95% CI)*	*p*
		High expression	Low expression		
Age (years)				0.98(0.92, 1.05)	0.648
< 60	37(46.25)	22	29		
≥ 60	43(53.75)	18	11		
Gender				1.41(0.07, 28.51)	0.819
Male	77(96.25)	38	39		
Female	3(3.75)	2	1		
Smoking status				5.22(1.46, 18.68)	**0.011**
Smoker	63(78.75)	28	34		
Non-smoker	17(21.25)	12	6		
Clinical stage				2.38(0.91, 6.26)	**0.077**
I-II	49(61.25)	20	29		
III-IV	31(38.75)	20	11		
Differentiation					
high	13(16.25)	7	6		
Moderatly	54(67.50)	27	27	0.726(0.13, 3.97)	0.712
Poorly	13(16.25)	6	7	0.802(0.22, 2.86)	0.734
Lymph node metastasis				1.34(0.51, 3.05)	0.555
Yes	35(43.75)	20	15		
No	45(56.25)	20	25		

### LncRNA *SFTA1P* decreased cells viability and induced cell apoptosis

**Figure 3:**
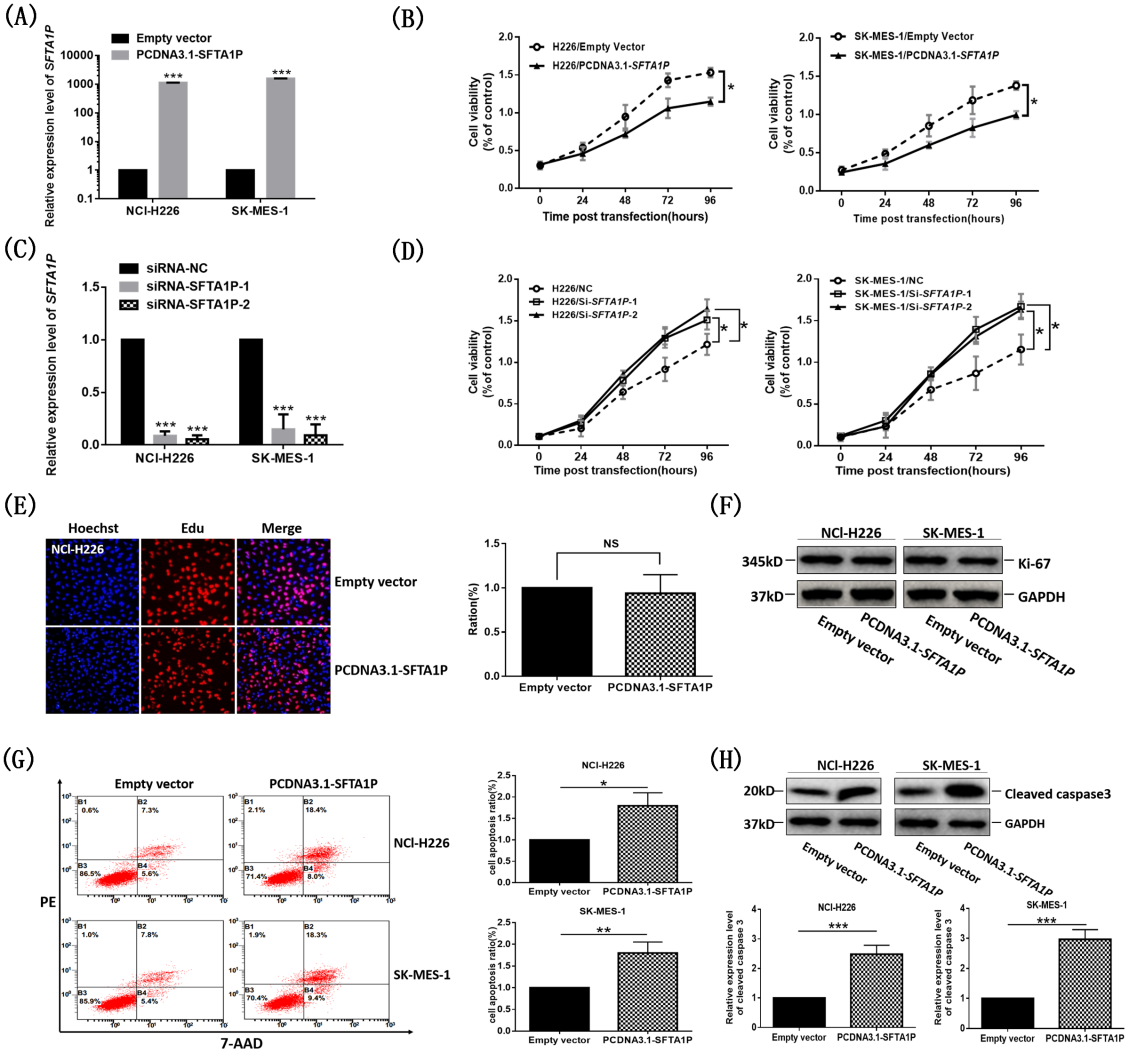
The cell viability and apoptosis induction in NCl-H226 and SK-MES-1 cells with elevated expression The *SFTA1P* expression level was detected by qPCR after overexpression **(Panel A)** or knockdown **(Panel C)** *SFTA1P* 24h in NCl-H226 and SK-MES-1 cells. **Panel B** and **Panel D.** NCl-H226 and SK-MES-1 cells growth were measured by MTS assay. **Panel E.** The effect of *SFTA1P* on cell proliferation was measured by Edu staining assay in NCl-H226 cells. NCl-H226 cells were cultured in 96-well plates and transfected with 1μg PCDNA3.1-*SFTA1P* vector or empty vector for 24h, and then exposed to EdU (10uM) for 12h. Proliferating of NCl-H226 cells were labeled with Edu (red) and DAPI (blue) to visualize the nuclei. The EdU-labeled replicating cells were examined under a fluorescent microscope. Data shown are representative images of individual groups (n=6 per group) from three independent experiments. Note that the number of NCl-H226 cells that incorporated EdU during transfected with PCDNA3.1(+)-*SFTA1P* vector did not significantly increase. **Panel F.** Western blot analysis of cell proliferation marker protein Ki-67 in *SFTA1P*-overexpression and control NCl-H226 and SK-MES-1 cells post transfection 48h. **Panel G.** 7-ADD/PE staining and flow cytometry analysis showed that *SFTA1P* overexpression in NCl-H226 and SK-MES-1 cells dramatically enhanced the cell apoptosis rate compared with control groups. **Panel H.** Western blot analysis of cell apoptosis marker protein cleaved caspase3 in *SFTA1P*-overexpression and control NCl-H226 and SK-MES-1 cells post transfection 48h. All experiments were performed in three biological replicates (n =3). And all data are shown as mean ±SD, ^*^p<0.05, ^**^p<0.01, ^***^p<0.001.).

To assess the biological function of *SFTA1P* on LSCC phenotype, we performed gain of-function assays to investigate the influence of *SFTA1P* on cell proliferation, apoptosis by transfecting PCDNA3.1(+)-*SFTA1P* vector into the LSCC cell lines (NCl-H226, SK-MES-1). Cells transfected with PCDNA3.1(+)-*SFTA1P* vector showed significantly increased *SFTA1P* expression in NCl-H226 and SK-MES-1 cells compared with that of the control group (Figure 3A). The MTS assay showed that the cell viability was decreased in *SFTA1P* overexpressing NCl-H226 and SK-MES-1 cells (Figure 3B). Oppositely, knockdown of *SFTA1P* by transfecting *SFTA1P*-specific siRNA (Figure 3C) led to increased cell viability (Figure 3D). Collectively, these results suggested that *SFTA1P* had a suppressive effect on LSCC cell viability possibly through inhibiting cell proliferation or inducing cell apoptosis.

EdU, an indicator of DNA synthesis, was used to detect the effect of *SFTA1P* on cell proliferation. Results showed that there was no significant difference between the percentage of EdU positive cells in the *SFTA1P* over-expression group and that of the control group (Figure 3E). In addition, we detected the cell proliferation marker protein Ki-67 expression level in NCl-H226 and SK-MES-1 cells, it also showed the same results (Figure 3F). These results suggest that *SFTA1P* has no effect on LSCC cell proliferation. So, we guessed that *SFTA1P* decreased cell viability probably via inducing cell apoptosis.

Then, we performed flow cytometric analysis to detect whether *SFTA1P* inducing cell apoptosis. Our results showed that the fraction of apoptotic cells was significantly increased in the *SFTA1P* overexpressing cells (Figure 3G). And, these results were further verified by Western blot analysis of cell apoptosis marker protein cleaved caspase3 (Figure 3H). It confirmed that *SFTA1P* was involved in the apoptosis of LSCC cells.

### Association of LncRNA *SFTA1P* with cell apoptosis and cisplatin-sensitivity

As illustrated in Figure 2C, *SFTA1P* expression was downregulated in cisplatin resistant lung adenocarcinoma cell line A549/DDP, as compared with that in the parental cell line A549, suggesting that *SFTA1P* may involve in cisplatin chemoresistance for lung cancer.

**Figure 4:**
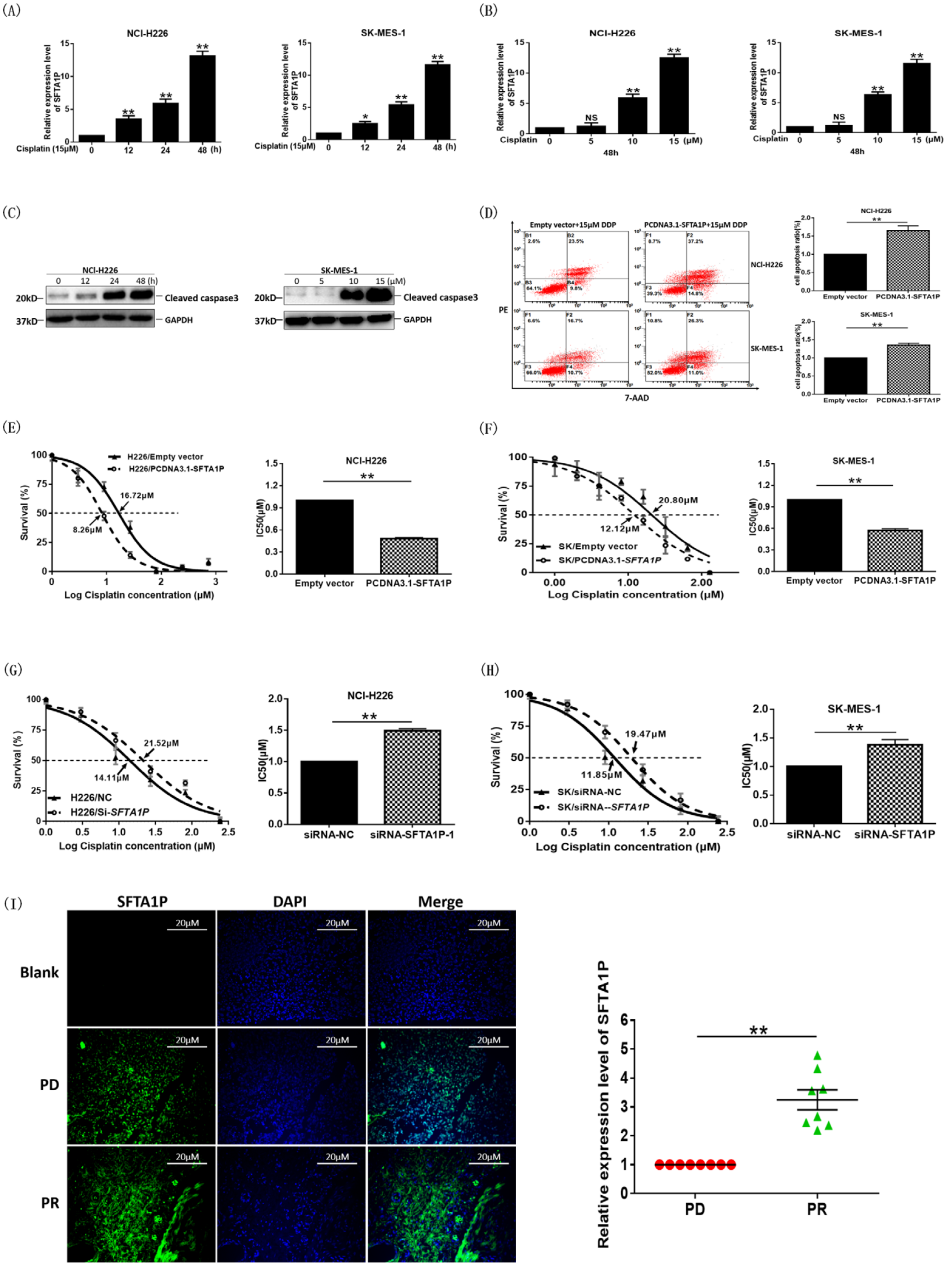
The cisplatin sensitivity was enhanced in LSCC cell lines and tissues while FTA1P was induced by cisplatin **Panel A.** NCl-H226 and SK-MES-1 cells were treated with 15μΜ cisplatin for the indicated times. The mRNA level of *SFTA1P* was detected by q-RT-PCR. **Panel B.** NCl-H226 and SK-MES-1 cells were treated with cisplatin at the indicated doses for 48h, and then, the alteration of *SFTA1P* levels was analyzed by q-RT-PCR. **Panel C.** Apoptosis was detected by western blot analysis. After treated with cisplatin at the indicated doses and for the indicated times, NCl-H226 cell lysates were analyzed by western blot analysis using the indicated antibodies. **Panel D.** Cell apoptosis was also evaluated using 7-ADD/PE staining with flow cytometry after the treatment with 15μM cisplatin for 24h in NCl-H226 and SK-MES-1 cell lines. Representative flow cytometry results showing *SFTA1P* effects on cisplatin induced cell apoptosis in NCl-H226 and SK-MES-1 cells. **Panel E-H.** NCl-H226 and SK-MES-1 cells overexpressed or knockdown *SFTA1P* were treated with different concentrations of cisplatin (1μM to 256μM). 48h later, cell viability was tested with an MTS assay. The half maximal inhibitory concentration (IC_50_) was calculated from 3 independent experiments using GraphPad 5.0 software. The overexpression *SFTA1P* group exhibited a lower IC_50_ of cisplatin than the empty vector group. The data represent mean ± SD of three independent experiments. ^*^p < 0.05, ^**^p < 0.01. **Panel I.** RNA fluorescent *in situ* hybridization (FISH) analysis of *SFTA1P* expression in LSCC biopsy formalin-fixed paraffin-embedded (FFPE) tissues with cisplatin chemotherapy curative effect evaluation (n=16). Cell nuclei are dyed DAPI shown in blue and *SFTA1P*-specific FISH shown in green. The first row represents the blank control, the second row represents the patients were resistance to cisplatin (PD), and the third row represents the patients were sensitive to cisplatin (PR).

In order to explore whether *SFTA1P* is essential for cisplatin chemosensitivity in LSCC, we determined the *SFTA1P* expression levels either at different time points in NCl-H226 and SK-MES-1 cells treated with 15μΜ cisplatin (Figure 4A) or 48h after cells were treated with cisplatin at the indicated doses (Figure 4B). Interestingly, we observed that cisplatin induced *SFTA1P* expression in a time-dependent and dose-dependent manner. Meanwhile, we noticed that the cell apoptosis was also increased in a dose-dependent manner by detecting the apoptosis marker protein cleaved caspase 3 (Figure 4C). These data indicated that *SFTA1P* may involve in cisplatin chemosensitivity in LSCC.

Furthermore, cisplatin sensitivity, we performed MTS and flow cytometry assays to assess cell viability and apoptosis upon cisplatin treatment, respectively. Flow cytometric analyses using PE and Annexin V7-ADD double staining showed that when cells were treated with 15μM cisplatin for 24h, the fraction of apoptotic cells was notably increased in *SFTA1P*-overexpressing NCl-H226 cells compared with cells transfected with empty vector (Figure 4D).

IC_50_ value for cisplatin of cells overexpressing *SFTA1P* or with *SFTA1P* depletion was measured. Compared with the control groups, the IC_50_ value for cisplatin in NCl-H226 (or SK-MES-1) transfected with *SFTA1P* expressing vector was significantly reduced (Figure 4E and 4F). Conversely, compared with NCl-H226 (or SK-MES-1) cells transfected with siRNA-NC, the IC50 value for cisplatin in NCl-H226 or SK-MES-1 transfected with *SFTA1P*-specific siRNA was significantly increased (Figure 4G and 4H). These results demonstrated that LncRNA *SFTA1P* enhanced sensitivity of LSCC cell lines to cisplatin.

Further, to confirm the role of *SFTA1P*, we used RNA fluorescence *in situ* hybridization (FISH) analysis to detect *SFTA1P* expression levels in 16 formalin-fixed paraffin-embedded (FFPE) LSCC tissues from patients treated with cisplatin-based chemotherapy. Eight patients experienced PR (partial response) and eight patients experienced PD (progressive disease) according to the RECIST criteria (Response Evaluation Criteria in Solid Tumors). As shown in Figure 4I, *SFTA1P* expression was significantly higher in the PR patients than in the PD patients. These results demonstrated that lncRNA *SFTA1P* was possibly involved in the chemoresistance to cisplatin.

### LncRNA *SFTA1P* upregulated the expression of hnRNP-U

**Figure 5:**
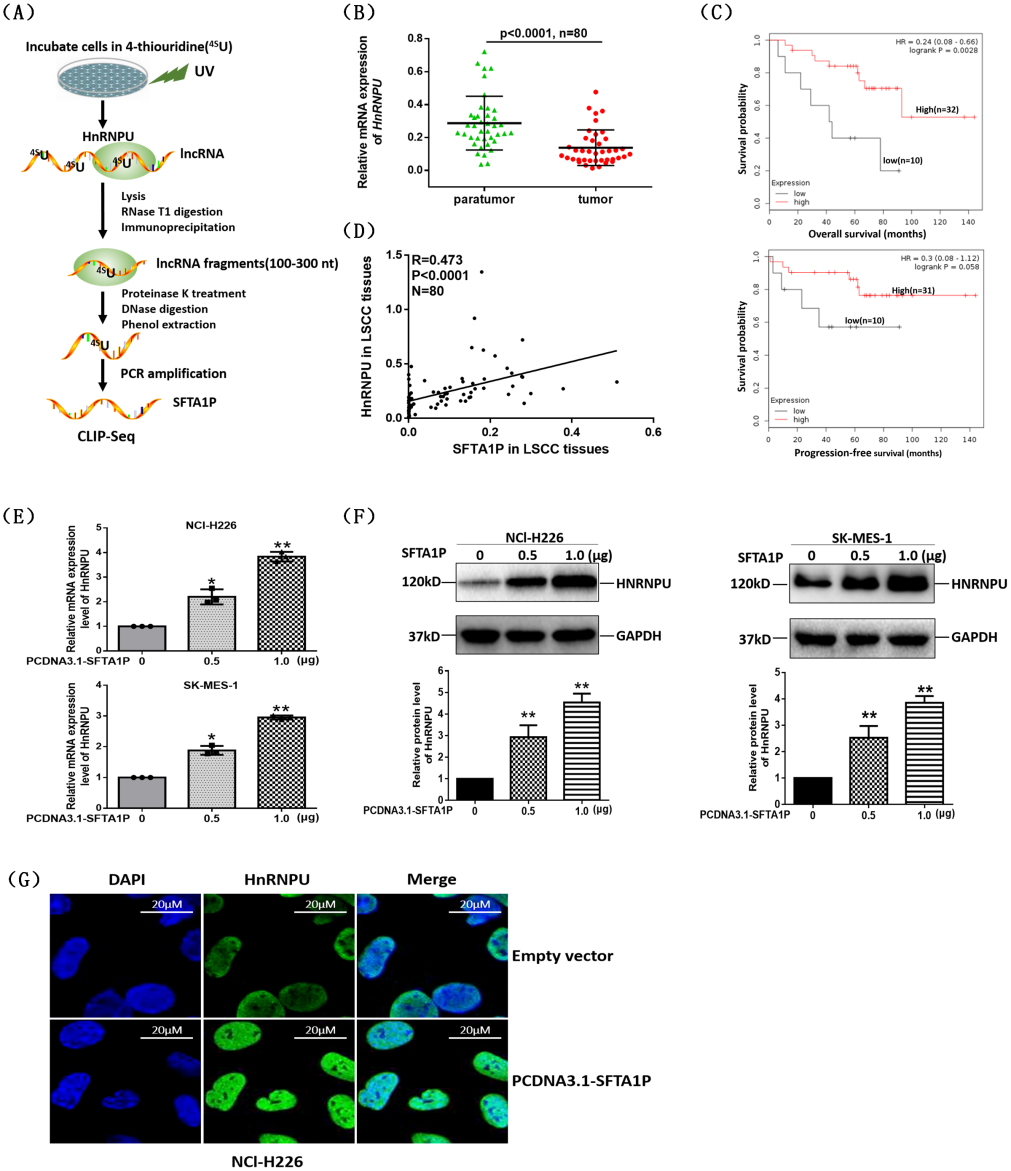
LncRNA *SFTA1P* upregulated the expression of hnRNP-U **Panel A.** Schematic representation of the multiple steps of CLIP-seq. CLIP-seq analysis identified *SFTA1P* targets bound by hnRNPU (data from GSE34993). **Panel B.** Relative mRNA expression of hnRNPU in LSCC tissues detected by qRT-PCR. **Panel C.** Kaplan-Meier analysis of overall survival (OS) and progression-free survival (PFS) in LSCC patients (data from GSE8894), and the median hnRNPU expression was used as a cutoff. It shows that high level of hnRNPU expression were correlated with a good OS and PFS. **Panel D.** The correlation between hnRNPU mRNA and *SFTA1P* expression in LSCC tumor tissues and para-tumor tissues (n=80). **Panel E** and **Panel F.** After respectively transfected 0.5μg and1.0μg PCDNA3.1-*SFTA1P* vector for 24h or 48h, qRT-PCR and Western blot analysis were used to detecte the hnRNPU expression in NCl-H226 or SK-MES-1. Results are presented as mean ± SD, (n=3). ^**^p< 0.01. **Panel G.** The immumofluorescence observation (100×) of hnRNPU (green) in NCl-H226 cells after transfected with PCDNA3.1 (+)-*SFTA1P* plasmid or empty vector for 48h. It shows that hnRNPU located in the nucleus. And the overexpression *SFTA1P* group exhibited a higher expression of hnRNPU than the empty vector group.

We further explored the potential mechanism how *SFTA1P* influence cisplatin-induced apoptosis and regulate cisplatin resistance in LSCC. Through analyzing the data from a previous study (GSE34993) [26], we found that *SFTA1P* binded with heterogeneous nuclear ribonucleoprotein U (hnRNP-U, also called scaffold attachment factor A (SAF-A)) based on crosslinking and immunoprecipitation coupled with high-throughput sequencing (CLIP-seq) (p=1.12E-44, hypergeometric test; Figure 5A). We next examined RNA expression levels of *SFTA1P* and hnRNP-U in LSCC and adjacent normal tissues (n=80). As shown in Figure 5B and 5C, hnRNP-U expression was reduced in LSCC tissues compared with tissues. And further Kaplan–Meier survival analysis showed that LSCC patients with high hnRNP-U expression had notably longer overall survival (OS) and progression-free survival (PFS) than those with low hnRNP-U expression in GSE50081 dataset (Figure 5C). Moreover, we observed a significantly positive correlation between the mRNA level of hnRNP-U and *SFTA1P* transcriptional level in LSCC tissues (Figure 5D), which further suggests that upregulation of *SFTA1P* may lead to elevated expression of hnRNP-U in LSCC.

To determine the effect of *SFTA1P* on hnRNP-U expression, we detected hnRNP-U mRNA and protein alteration in NCl-H226 and SK-MES-1 cell lines transfected with *SFTA1P* overexpressing vector. The results showed that *SFTA1P* expression significantly increased hnRNP-U mRNA and protein levels in a dose-dependent manner (Figure 5E and 5F). This phenomenon was also observed by immune-fluorescence assay (Figure 5G).

### LncRNA *SFTA1P* and hnRNP-U expression were correlated with GADD45A expression in LSCC cells and tissues

HnRNP-U is an abundant nuclear protein. It belongs to the subfamily of ubiquitously expressed heterogeneous nuclear ribonucleoproteins (hnRNPs). It was essential for cell viability [27], RNA stability control [20], and DNA damage response [23, 28].

**Figure 6:**
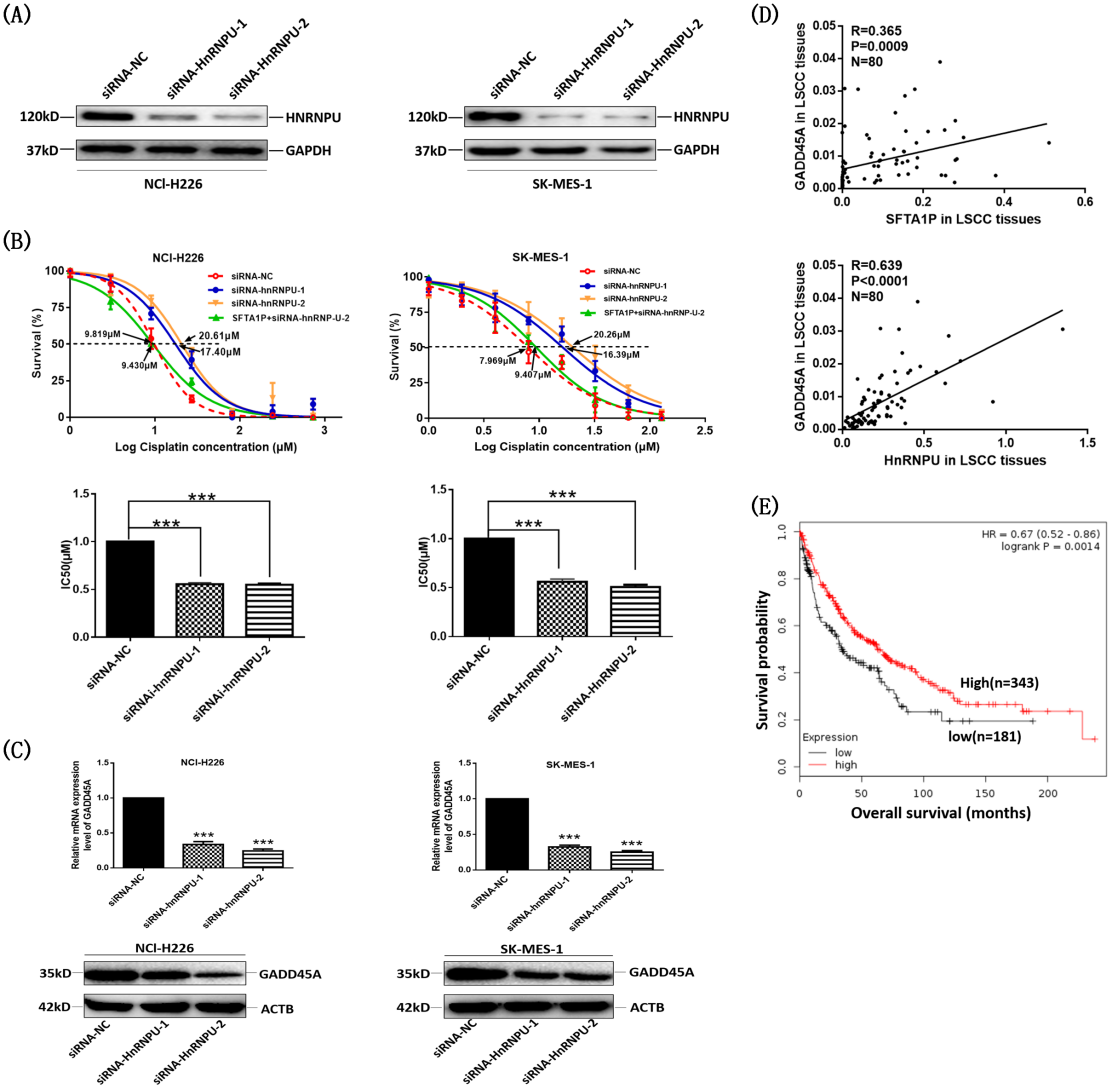
LncRNA *SFTA1P* and hnRNP-U expression were correlated with GADD45A expression in LSCC cells and tissues **Panel A.** Knockdown of hnRNPU was checked by Western blot in NCl-H226 andSK-MES-1 cells after transfection 24h. **Panel B.** After transfected 24h, NCl-H226 and SK-MES-1 cells were treated with different concentrations of cisplatin, and then cell viability was tested with an MTS assay. 48h later, the half maximal inhibitory concentration (IC_50_) was calculated from 3 independent experiments using GraphPad 5.0 software. The data represent mean ± SD of three independent experiments. ^***^p < 0.001. **Panel C.** After knockdown hnRNPU 24h or 48h, the mRNA and protein of GADD45A were detected by qRT-PCR and Western blot analysis. **Panel D.** The correlation between hnRNPU, GADD45A mRNA and *SFTA1P* expression in LSCC tumor tissues and para-tumor tissues (n=80). **Panel E.** Kaplan-Meier analysis of overall survival (OS) in LSCC patients (http://kmplot.com/analysis/index.php?p=service&start=1), and the median GADD45A expression was used as a cutoff. It shows that high level of GADD45A expression was correlated with a good OS.

To investigate the biological functions of hnRNP-U in the cisplatin sensitivity of LSCC, we performed MTS assay to examine the effect of hnRNP-U on cell sensitivity to cisplatin in NCl-H226 and SK-MES-1 cells. Cells were transfected with hnRNP-U-specific siRNA and the protein expression of hnRNP-U was detected by western blot (Figure 6A). IC_50_ value for cisplatin after hnRNP-U down-regulation was measured. Compared with NCl-H226 (or SK-MES-1) cells transfected with siRNA-NC, the IC_50_ value for cisplatin in NCl-H226 (or SK-MES-1) transfected with siRNA-hnRNP-U-1 and siRNA-hnRNP-U-2 was significantly increased (Figure 6B). While co-transfected PCDNA3.1-*SFTA1P* and siRNA-hnRNP-U-2 in LSCC cell lines, the effect of *SFTA1P* induced sensitivity to cisplatin was abolished (Figure 6B). Together, these data suggest that *SFTA1P* induces apoptosis and increases LSCC cells sensitivity to cisplatin through upregulating hnRNP-U expression.

A previous study has shown that hnRNP-U can enhance the expression of growth arrest and DNA damage-45 alpha (GADD45A) by stabilizing its mRNA [20]. GADD45A is often up-regulated in response to various environmental stresses and drug therapies [29, 30]. It has been shown that GADD45A plays a role in inhibiting cell growth [31], promoting apoptosis [32], participating in DNA repair, and cancer cell survival.

We further identified the underlying mechanism by which hnRNP-U regulates GADD45A expression using qPCR and WB. As shown in Figure 6C, after hnRNP-U knockdown, GADD45A mRNA and protein expression was significantly down-regulated. Meanwhile, our correlation analysis found that *SFTA1P* and hnRNP-U expression was positively correlated with GADD45A level in 80 LSCC tissues (Figure 6D). Further Kaplan–Meier survival analysis illustrated that patients with low GADD45A expression had notably shorter overall survival (OS) (Figure 6E). These results suggest that *SFTA1P* and hnRNP-U contributed to cisplatin sensitivity in LSCC via induction of GADD45A expression.

## DISCUSSION

Cisplatin is an important drug used in LSCC chemotherapy [3, 4]. However, the chemoresistance impedes its clinical usage [33, 34]. Chemoresistance remains a major obstacle in LSCC chemotherapy. Therefore, a better understanding of the underlying molecular mechanisms of tumourigenesis and chemoresistance is urgently required for improving LSCC treatment.

LncRNAs are a class of ncRNAs with transcript size >200 nucleotides [35]. They resemble mRNAs structurally but display a highly tissue-specifc expression pattern [36]. So, lncRNAs provide an opportunity to identify both cancer-specific biomarkers and functional drivers for lung cancer. Accumulating studies have demonstrated that lncRNAs play an important role in tumourigenesis and chemoresistance. For example, long noncoding RNA CCAT1 was reported to act as an oncogene and promote chemoresistance in docetaxel-resistant lung adenocarcinoma cells [37]; lncRNA FENDRR may act as an inhibitory molecule of doxorubicin-resistance through down-regulating the expression of ABCB1 and ABCC1 genes in osteosarcoma cells [38]; long non-coding RNA LINC00161 has been showed to sensitize osteosarcoma cells to cisplatin-induced apoptosis by regulating the miR-645-IFIT2 axis [39]. However, our knowledge of tumourigenesis and cisplatin chemosensitivity related lncRNAs in LSCC is still limited.

In our previous study, we investigated the genome-wide expression profile of lncRNAs in 16 LSCC tumors and matched adjacent normal lung tissues by microarray. A total of 852 down- and 2,748 up-regulated probes were identified to be significantly differentially expressed in tumor tissues [18]. Among these, we identified that *SFTA1P* expression was significantly decreased in LSCC tumor tissues compared with matched adjacent normal lung tissues (Figure 2A) and was correlated with poor prognosis (Figure 2D and 2E). Furthermore, we found that lncRNA *SFTA1P* expression was highly tissue specific in lung (Figure 1F), and it was downregulated in cisplatin-resistant cells (A549-DDP) (Figure 2C). Similar results were obtained in cisplatin-resistant LSCC patient’s tumor tissues (Figure 4I). In addition, we found that *SFTA1P* could be induced by cisplatin (Figure 4A and 4B). We further used gene intervention technology to overexpress *SFTA1P* and found that cell apoptosis and sensitivity to cisplatin was markedly increased, and vise verse. Overexpression of *SFTA1P* can improve the sensitivity of LSCC cells to cisplatin, which demonstrated that *SFTA1P* was possibly involved in chemoresistance and may serve as a biomarker to predict the chemo-response and prognosis of LSCC patients (Figures 3G and 4D-4H). The molecular mechanism that leads to the altered levels of *SFTA1P* during cisplatin treatment will be investigated in future.

Subsequently, we explored the potential mechanism of *SFTA1P* in response to cisplatin treatment in LSCC. By analyzing the crosslinking and immunoprecipitation combined with high-throughput sequencing (CLIP-seq) data (from GSE34993), we found that *SFTA1P* could bind with hnRNP-U. HnRNP-U has been reported to play a crucial role in various pathological and physiological processes such as cell viability [27], RNA stability control [20], and DNA damage response [23, 28]. In the current study, we found that *SFTA1P* increased cisplatin sensitivity through up-regulating the expression of hnRNP-U in LSCC (Figures 5E-5G and 6B). However, the underlying regulatory mechanism remains unclear.

It is well known that lncRNAs molecular function depends on their subcellular localization. In this study, we detected the subcellular localization of *SFTA1P* by FISH assay and cytoplasmic and nuclear RNA fractionation analysis, we found that *SFTA1P* was mainly located in the nucleus (Figure 1D and 1E). It is widely accepted that lncRNAs locating in nucleus have regulatory roles in gene expression at the transcriptional levels. According to the experiment results, we suspect that *SFTA1P* regulated the hnRNP-U expression transcriptionally, but the specific regulation mechanisms still need to be further validated.

It has shown that hnRNP-U can enhance the expression of GADD45A by stabilizing mRNA [20]. GADD45 is involved in the DNA damage repair [31], and promoting apoptosis [32, 40], it is also essential for cancer cell survival [32]. Consistently, we further found that the expression of *SFTA1P* was significantly positively correlated with hnRNP-U and GADD45A, and high expression of hnRNP-U, GADD45A contributed to longer OS in LSCC patients. Further PCR and WB assays showed that *SFTA1P* enhanced hnRNP-U expression and promoted apoptosis. On the basis of the previously reported mechanism by which hnRNP-U regulates gene expression, we hypothesized that it promoted the expression of GADD45A. And it was validated by down-regulation of hnRNP-U (Figure 6). However, the specific regulatory mechanism between lncRNA SFTA1P and these two drug resistance related genes needs to be further studied by RNA pull down and chromatin immunoprecipitation.

In summary, we have shown that lncRNA *SFTA1P* and hnRNP-U were down-regulated in LSCC tissues and correlated with poor prognosis in LSCC patients. Both of them are involved in the progression of cisplatin sensitivity in LSCC. *SFTA1P* expression could be induceed by cisplatin and it promoted apoptosis and enhanced cisplatin sensitivity in LSCC by upregulating the expression of hnRNP-U at both the mRNA and protein levels. Moreover, *SFTA1P* facilitated the DNA damage repair and apoptosis related gene GADD45A expression. Our results indicate that lncRNA *SFTA1P* sensitizes LSCC cells to cisplatin-induced apoptosis by regulating the hnRNP-U-GADD45A axis, and it might be a candidate prognostic biomarker and a target for reversing cisplatin resistance in LSCC.

## MATERIALS AND METHODS

### Tissue specimens’ collection

We obtained 80 LSCC tissues and paired adjacent non-tumor lung tissues (NTL) from patients who underwent surgery and 16 formalin-fixed paraffin-embedded (FFPE) tissues from patients that were diagnosed with LSCC based on histopathological evaluation at Xiangya Hospital of Central South University (Changsha, Hunan, China). All of them provided written informed consents, in compliance with the code of ethics of the World Medical Association (Declaration of Helsinki) at the time of surgery for the donation of their tissues for this research. This project’s protocol was approved by the Ethics Committee of Xiangya School of Medicine, Central South University (registration number of CTXY-110008-3). All fresh tissues were instantly frozen in liquid nitrogen after resection and stored at -80°C until use, and the FFPE tissues were stored in a cool, dry place at room temperature.

### Cell culture and reagents

Five non-small cell lung cancer cell lines (NCl-H226, SK-MES-1, NCl-H1299, A549, A549-DDP (cisplatin resistance cell line)) and a normal bronchial epithelial cell line HBE were obtained from the Institute of Biochemistry and Cell Biology of the Chinese Academy of Sciences (Shanghai, China). NCl-H226, NCl-H1299, A549 and A549-DDP cells were cultured in RPMI-1640 containing 10% fetal bovine serum (FBS, Gibico-BRL), and HBE, SK-MES-1 cells were cultured with DMEM and MEM, respectively. Medium was renewed every day and cells were passaged before reaching confluence.

### RNA extraction and real-time RT-PCR

To isolate cytoplasmic and nuclear RNAs from cells, we used a PARIS kit (Ambion) and followed the manufacturer’s instruction. Total RNA was isolated using Trizol (Invitrogen) from tissue samples or cells according to the manufacturer’s instructions. One microgram of total RNA was reverse transcribed in a final volume of 20μl using a PrimeScriptTM RT reagent kit (Takara, RR091A) according to the manufacturer’s instructions. Quantitative reverse transcriptase polymerase chain reaction (qRT-PCR) was performed in triplicate on the LC480, and data were calculated using the 2^-ΔΔCT^ method. All primer sequences were listed in Supplementary Table 1.

### Cell transfection

**Overexpression**: The human *SFTA1P* complementary DNA (cDNA) sequence was synthesized and then subcloned into the pCDNA3.1 (+) vector (GeneChem, Shanghai, China) to generate the pCDNA3.1 (+)-*SFTA1P* vector. Empty vector was used as a control.

**Knockdown**: Silencing of *SFTA1P* was achieved by specific siRNAs. Three *SFTA1P*-specific siRNAs were synthesized by Ribobio (Guangzhou, China), the siRNA sequences were listed in Supplementary Table 1. A scrambled siRNA was used as negative control (NC). NCl-H226, SK-MES-1 cells were transfected with pCDNA3.1 (+)-*SFTA1P* vector, empty vector and siRNAs using Lipofectamine 2000 (Invitrogen, Carlsbad, CA, USA) according to the manufacturer’s directions, and the cells were incubated for 24h before experimental use.

### Drug sensitivity assay

Cells were seeded in 96-well plates at a proper density of 4000 cells per well and were incubated overnight at 37°C. The cells were incubated with different concentrations of cisplatin for 48h at 37°C. After addition of 20μl of Cell Titer 96^®^ AQueous One Solution Cell Proliferation Assay (MTS) (Promega) to each well, plates were incubated for 1h at 37°C. Absorbance of each well at 490nm (A490) was detected using a spectrophotometer. The concentration of each drug that gives rise to 50% inhibition of growth (IC_50_) was estimated from relative survival curves. Three independent experiments were performed in six duplicate wells. Cisplatin was obtained from Sigma-Aldrich (St. Louis, MO, USA, P4394).

### EdU incorporation assay

The impact of overexpressing *SFTA1P* on NCl-H226 and SK-MES-1 cells proliferation was measured by 5-ethynyl-20-deoxyuridine (EdU) incorporation assay using an EdU assay kit (Cat.No:C10310, RiboBio, China). 24h after transfection, cells were incubated with culture medium supplemented with 10μM EdU for 12h. And then, cells were treated following the manufacturer’s instruction.

### Apoptosis detection by flow cytometry assay

NCl-H226 and SK-MES-1 cells were seeded in six-well plates. After growing for 12h, cells were transiently transfected with the PCDNA3.1-*SFTA1P* or empty vector and harvested by trypsinization 48h later, or treated with 15μM cisplatin for 24h followed by resuspension in complete medium. Next, cells were washed twice with cold PBS and resuspended in 1X Binding Buffer at a concentration of 1 x 10^6^ cells/ml. Then, cells were treated following the manufacturer’s instruction of PE Annexin V Apoptosis Detection Kit I (BD, Cat. NO: 559763). The procedures were described as follows: Transfer 100 μl of the solution (1x10^5^ cells) to a 5ml culture tube, then add 5μl of PE Annexin V and 5μl 7-AAD, gently vortex the cells and incubate for 15 min at RT (25°C) in the dark, finally add 400μl of 1X Binding Buffer to each tube. Analyze by flow cytometry within 1h.

### RNA fluorescence *in situ* hybridization (FISH)

The *SFTA1P* cDNA probes were designed and synthesized by BersinBio (Guangzhou, China). These probes (probe A: 1-TTCCCAGGGCGCTCTCTGCCAA G-23; probe B: 390-TTTCCATCCTGAGGT GGTCTGCC-412; probe C: 849-AAG ATGAATGTAAGGTTTTATTG-871) were labeled by 5’-FAM. Then, the RNA FISH experiments were performed on NCl-H226, A549 cells and on paraffin embedded sections of 8 LSCC and their adjacent tissues. The RNA FISH was then carried out following the instruction of the FISH Detection Kit (BersinBio, Guangdong, China). Images were taken using a fluorescence microscope equipped with vision software. Detected signals were colored green (*SFTA1P*) and blue (DAPI). The fluorescence signals were quantified by image J software.

### Immunofluorescence detection

HnRNPU protein expression alteration in NCl-H226 cells was observed by immunofluorescence 48h after cells were transfected with *SFTA1P* overexpressing vector. After growing for 12h, cells transiently transfected with the PCDNA3.1-*SFTA1P* or empty vectors were inoculated in special petri dish. After transfected for 48h, the cells were fixed with 4% paraformaldehyde, then washed in PBS at room temperature 3 times. Then the cells were permeabilized with 0.1% Triton X-100 (Beyotime) in PBS for 30min at room temperature, then washed in PBS three times. After incubated for 30min, 1% BSA incubated in PBST was used to block the binding of non-specific sites. 60 min later, primary antibody of hnRNPU (1:100, ab10297) in 1% BSA was added and the cells were incubated for 60min at room temperature. Then 20μl fluorescence labeling antibody (1:50, Beyotime) was added and incubated for 30min. Nuclei were stained with DAPI (Cat. No A1001, Applichem, Germany) at a concentration of 10 ng/ml in cold dark place for 15 min. Finally, cells were observed by fluorescent microscope.

### Western blot analysis

48h after transfection, cells were collected and lysed using a RIPA lysis buffer that contained the protease inhibitor cocktail (Roche, Basel, Switzerland) and phenyl methyl sulfonyl fluoride (PMSF) (Roche). Cell protein lysates that contained 50μg protein were electrophoresed on 10% or 12% sodium dodecyl sulfate-polyacrylamide gel, and then transferred onto 0.22mm or 0.45mm PVDF membrane (Millipore, Bedford, MA). Then, the membranes were incubated in blocking solution (5% non-fat milk) for 1.5 hour at room temperature (RT) and incubated with specific primary antibodies at 4°C overnight. The following antibodies were used in this study: GAPDH (Sigma, G9545); ACTB (Beyotime, AA128); Cleaved Caspase-3 (Wanleibio, WL02348); Ki-67 (Wanleibio, WL01384a); GADD45A (Sigma, WH00016 47M1).

### Statistical analysis

The statistical analyses were performed using SPSS 20.0 software (IBM, Inc., Chicago, IL, USA). The significance of differences between groups was estimated by the Student’s t-test, or chi-square test. Pearson correlation analyses were conducted to investigate the correlation between *SFTA1P* and other gene expressions. Two-sided p values were calculated, and a value of P<0.05 was considered statistically significance. All values were presented as the mean ± SD.

## SUPPLEMENTARY MATERIALS TABLE





## References

[1] Hammerman PS, Lawrence MS, Voet D, Jing R, Cibulskis K, Sivachenko A, Stojanov P, McKenna A, Lander ES, Gabriel S, Getz G, Sougnez C, Imielinski M (2012). Comprehensive genomic characterization of squamous cell lung cancers. Nature.

[2] Crino L, Weder W, van Meerbeeck J, Felip E (2010). Early stage and locally advanced (non-metastatic) non-small-cell lung cancer: ESMO Clinical Practice Guidelines for diagnosis, treatment and follow-up. Ann Oncol.

[3] Scagliotti GV, Novello S, Rapetti S, Papotti M (2013). Current state-of-the-art therapy for advanced squamous cell lung cancer. Am Soc Clin Oncol Educ Book.

[4] Azzoli CG, Temin S, Aliff T, Baker SJ, Brahmer J, Johnson DH, Laskin JL, Masters G, Milton D, Nordquist L, Pao W, Pfister DG, Piantadosi S (2011). 2011 Focused update of 2009 American Society of Clinical Oncology Clinical Practice Guideline update on chemotherapy for stage IV non-small-cell lung cancer. J Clin Oncol.

[5] Schiller JH, Harrington D, Belani CP, Langer C, Sandler A, Krook J, Zhu J, Johnson DH (2002). Comparison of four chemotherapy regimens for advanced non-small-cell lung cancer. N Engl J Med.

[6] Wang J, Zhang J, Zheng H, Li J, Liu D, Li H, Samudrala R, Yu J, Wong GK (2004). Mouse transcriptome: neutral evolution of 'non-coding' complementary DNAs. Nature.

[7] Struhl K (2007). Transcriptional noise and the fidelity of initiation by RNA polymerase II. Nat Struct Mol Biol.

[8] Perkel JM (2013). Visiting “noncodarnia”. Biotechniques.

[9] Matsumoto A, Pasut A, Matsumoto M, Yamashita R, Fung J, Monteleone E, Saghatelian A, Nakayama KI, Clohessy JG, Pandolfi PP (2017). mTORC1 and muscle regeneration are regulated by the LINC00961-encoded SPAR polypeptide. Nature.

[10] Wang KC, Chang HY (2011). Molecular mechanisms of long noncoding RNAs. Mol Cell.

[11] Mercer TR, Dinger ME, Mattick JS (2009). Long non-coding RNAs: insights into functions. Nat Rev Genet.

[12] Bhartiya D, Kapoor S, Jalali S, Sati S, Kaushik K, Sachidanandan C, Sivasubbu S, Scaria V (2012). Conceptual approaches for lncRNA drug discovery and future strategies. Expert Opin Drug Discov.

[13] Qu L, Ding J, Chen C, Wu ZJ, Liu B, Gao Y, Chen W, Liu F, Sun W, Li XF, Wang X, Wang Y, Xu ZY (2016). Exosome-transmitted lncARSR promotes sunitinib resistance in renal cancer by acting as a competing endogenous RNA. Cancer Cell.

[14] Gong WJ, Yin JY, Li XP, Fang C, Xiao D, Zhang W, Zhou HH, Li X, Liu ZQ (2016). Association of well-characterized lung cancer lncRNA polymorphisms with lung cancer susceptibility and platinum-based chemotherapy response. Tumour Biol.

[15] Yan L, Yang M, Guo H, Yang L, Wu J, Li R, Liu P, Lian Y, Zheng X, Yan J, Huang J, Li M, Wu X (2013). Single-cell RNA-Seq profiling of human preimplantation embryos and embryonic stem cells. Nat Struct Mol Biol.

[16] Cabili MN, Trapnell C, Goff L, Koziol M, Tazon-Vega B, Regev A, Rinn JL (2011). Integrative annotation of human large intergenic noncoding RNAs reveals global properties and specific subclasses. Genes Dev.

[17] Huarte M (2015). The emerging role of lncRNAs in cancer. Nat Med.

[18] Wang Y, Qian CY, Li XP, Zhang Y, He H, Wang J, Chen J, Cui JJ, Liu R, Zhou H, Xiao L, Xu XJ, Zheng Y (2015). Genome-scale long noncoding RNA expression pattern in squamous cell lung cancer. Sci Rep.

[19] Fackelmayer FO, Richter A (1994). hnRNP-U/SAF-A is encoded by two differentially polyadenylated mRNAs in human cells. Biochim Biophys Acta.

[20] Yugami M, Kabe Y, Yamaguchi Y, Wada T, Handa H (2007). hnRNP-U enhances the expression of specific genes by stabilizing mRNA. FEBS Lett.

[21] Xiao R, Tang P, Yang B, Huang J, Zhou Y, Shao C, Li H, Sun H, Zhang Y, Fu XD (2012). Nuclear matrix factor hnRNP U/SAF-A exerts a global control of alternative splicing by regulating U2 snRNP maturation. Mol Cell.

[22] Gohring F, Schwab BL, Nicotera P, Leist M, Fackelmayer FO (1997). The novel SAR-binding domain of scaffold attachment factor A (SAF-A) is a target in apoptotic nuclear breakdown. EMBO J.

[23] Berglund FM, Clarke PR (2009). hnRNP-U is a specific DNA-dependent protein kinase substrate phosphorylated in response to DNA double-strand breaks. Biochem Biophys Res Commun.

[24] Lin MF, Jungreis I, Kellis M (2011). PhyloCSF: a comparative genomics method to distinguish protein coding and non-coding regions. Bioinformatics.

[25] Krupp M, Marquardt JU, Sahin U, Galle PR, Castle J, Teufel A (2012). RNA-Seq Atlas--a reference database for gene expression profiling in normal tissue by next-generation sequencing. Bioinformatics.

[26] Huelga SC, Vu AQ, Arnold JD, Liang TY, Liu PP, Yan BY, Donohue JP, Shiue L, Hoon S, Brenner S, Ares MJ, Yeo GW (2012). Integrative genome-wide analysis reveals cooperative regulation of alternative splicing by hnRNP proteins. Cell Rep.

[27] Roshon MJ, Ruley HE (2005). Hypomorphic mutation in hnRNP U results in post-implantation lethality. Transgenic Res.

[28] Britton S, Dernoncourt E, Delteil C, Froment C, Schiltz O, Salles B, Frit P, Calsou P (2014). DNA damage triggers SAF-A and RNA biogenesis factors exclusion from chromatin coupled to R-loops removal. Nucleic Acids Res.

[29] Hirose T, Sowa Y, Takahashi S, Saito S, Yasuda C, Shindo N, Furuichi K, Sakai T (2003). p53-independent induction of Gadd45 by histone deacetylase inhibitor: coordinate regulation by transcription factors Oct-1 and NF-Y. Oncogene.

[30] Yin F, Bruemmer D, Blaschke F, Hsueh WA, Law RE, Herle AJ (2004). Signaling pathways involved in induction of GADD45 gene expression and apoptosis by troglitazone in human MCF-7 breast carcinoma cells. Oncogene.

[31] Hollander MC, Fornace AJ (2002). Genomic instability, centrosome amplification, cell cycle checkpoints and Gadd45a. Oncogene.

[32] Zerbini LF, Wang Y, Czibere A, Correa RG, Cho JY, Ijiri K, Wei W, Joseph M, Gu X, Grall F, Goldring MB, Zhou JR, Libermann TA (2004). NF-kappa B-mediated repression of growth arrest- and DNA-damage-inducible proteins 45alpha and gamma is essential for cancer cell survival. Proc Natl Acad Sci U S A.

[33] Yang Y, Li H, Hou S, Hu B, Liu J, Wang J (2013). The noncoding RNA expression profile and the effect of lncRNA AK126698 on cisplatin resistance in non-small-cell lung cancer cell. PLoS One.

[34] Liu MY, Li XQ, Gao TH, Cui Y, Ma N, Zhou Y, Zhang GJ (2016). Elevated HOTAIR expression associated with cisplatin resistance in non-small cell lung cancer patients. J Thorac Dis.

[35] Ponting CP, Belgard TG (2010). Transcribed dark matter: meaning or myth?. Hum Mol Genet.

[36] Brunner AL, Beck AH, Edris B, Sweeney RT, Zhu SX, Li R, Montgomery K, Varma S, Gilks T, Guo X, Foley JW, Witten DM, Giacomini CP (2012). Transcriptional profiling of long non-coding RNAs and novel transcribed regions across a diverse panel of archived human cancers. Genome Biol.

[37] Chen J, Zhang K, Song H, Wang R, Chu X, Chen L (2016). Long noncoding RNA CCAT1 acts as an oncogene and promotes chemoresistance in docetaxel-resistant lung adenocarcinoma cells. Oncotarget.

[38] Kun-Peng Z, Xiao-Long M, Chun-Lin Z (2017). LncRNA FENDRR sensitizes doxorubicin-resistance of osteosarcoma cells through down-regulating ABCB1 and ABCC1. Oncotarget.

[39] Wang Y, Zhang L, Zheng X, Zhong W, Tian X, Yin B, Tian K, Zhang W (2016). Long non-coding RNA LINC00161 sensitises osteosarcoma cells to cisplatin-induced apoptosis by regulating the miR-645-IFIT2 axis. Cancer Lett.

[40] Zerbini LF, Wang Y, Correa RG, Cho JY, Libermann TA (2005). Blockage of NF-kappaB induces serine 15 phosphorylation of mutant p53 by JNK kinase in prostate cancer cells. Cell Cycle.

