# Supplementary material for: Long noncoding RNA SFTA1P promoted apoptosis and increased cisplatin chemosensitivity via regulating the hnRNP-U-GADD45A axis in lung squamous cell carcinoma
Source: Oncotarget. 2017 Oct 27;8(57):97476–89. doi: 10.18632/oncotarget.22138 (PMC5722577; doi:10.18632/oncotarget.22138)
Supplement: Supplementary file 1 [file oncotarget-08-97476-s001.pdf]

Long noncoding RNA *SFTA1P* promoted apoptosis and increased cisplatin chemosensitivity via regulating the hnRNP-U-GADD45A axis in lung squamous cell carcinoma

SUPPLEMENTARY MATERIALS

Supplementary Table 1: Primers and siRNA sequences used in this article

See Supplementary File 1
